# Gamma Oscillations and Potassium Channel Modulation in Schizophrenia: Targeting GABAergic Dysfunction

**DOI:** 10.1177/15500594221148643

**Published:** 2023-01-02

**Authors:** Stephen J. Kaar, Judith F. Nottage, Ilinca Angelescu, Tiago Reis Marques, Oliver D. Howes

**Affiliations:** 1 Department of Psychosis Studies, 34426 Institute of Psychiatry, Psychology & Neuroscience, King's College London, London, UK; 2 MRC London Institute of Medical Sciences, Hammersmith Hospital, London, UK; 3 Division of Psychology and Mental Health, Faculty of Biology, Medicine, and Health, University of Manchester, Manchester, UK; 4 Department of Neuroimaging, 34426 Institute of Psychiatry, Psychology & Neuroscience, King's College London, London, UK; 5 Max Planck UCL Centre for Computational Psychiatry and Ageing Research London, London, UK; 6 Faculty of Medicine, Institute of Clinical Sciences (ICS), Imperial College London, London, UK

**Keywords:** gamma oscillations, parvalbumin, psychosis, potassium channels, novel treatment

## Abstract

Impairments in gamma-aminobutyric acid (GABAergic) interneuron function lead to gamma power abnormalities and are thought to underlie symptoms in people with schizophrenia. Voltage-gated potassium 3.1 (Kv3.1) and 3.2 (Kv3.2) channels on GABAergic interneurons are critical to the generation of gamma oscillations suggesting that targeting Kv3.1/3.2 could augment GABAergic function and modulate gamma oscillation generation. Here, we studied the effect of a novel potassium Kv3.1/3.2 channel modulator, AUT00206, on resting state frontal gamma power in people with schizophrenia. We found a significant positive correlation between frontal resting gamma (35–45 Hz) power (*n* = 22, *r* = 0.613, *P* < .002) and positive and negative syndrome scale (PANSS) positive symptom severity. We also found a significant reduction in frontal gamma power (*t*_13_ = 3.635, *P* = .003) from baseline in patients who received AUT00206. This provides initial evidence that the Kv3.1/3.2 potassium channel modulator, AUT00206, may address gamma oscillation abnormalities in schizophrenia.

## Introduction

Schizophrenia is one of the leading causes of healthcare burden in the world,^1,2^ yet current antipsychotic treatments, all dopamine D2 receptor blockers, are inadequate for many patients, highlighting the need to develop alternative treatment approaches.^2,3^ Electroencephalography (EEG) studies show abnormalities in high-frequency electrical oscillations in the gamma frequency range (30 to 45 Hz) in people with schizophrenia relative to controls.^4–7^ Gamma oscillations are generated by the synchronised firing of large groups of excitatory pyramidal cells^8^ under inhibitory regulation from parvalbumin positive GABAergic interneurons.^9–13^ An imbalance in this excitatory and inhibitory signalling in people with schizophrenia is thought to underlie the abnormalities seen in gamma oscillations and is hypothesised to contribute to the symptoms seen in the disorder.^14–16^

Parvalbumin positive GABAergic interneurons (parvalbumin interneurons) are typically fast firing and form inhibitory synapses onto excitatory glutamatergic pyramidal neurons.^10^ They have been implicated in the pathophysiology of schizophrenia since the 1990s^17–26^ and multiple pre-clinical models indicate that parvalbumin interneuron pathology leads to phenotypes associated with schizophrenia.^27–33^ Suppression of parvalbumin interneuron activity in the pre-frontal cortex increases oscillation power in the gamma range,^30^ suggesting that deficiencies in parvalbumin interneurons could lead to the increased resting gamma power found in the disorder.^34^ In addition, patients with schizophrenia show lower availability of N-methyl-d-aspartate receptors (NMDA-R), and NMDA-R antagonists, such as ketamine, induce schizophrenia-like symptoms,^35–38^ and increase resting gamma power in healthy controls.^34^

Gamma-band activity is primarily distributed across frontotemporal regions^39,40^ and can be measured using several different electrophysiological paradigms including spontaneous activity at rest, as a response to task, or evoked by sensory stimuli.^34,41,42^ Excessive resting frontal gamma activity has been found in first-degree relatives of people with schizophrenia and has been proposed as a potential endophenotype and biomarker for cortical dysfunction in the disorder.^40,41,43,44^ Increased frontal resting gamma power has also been found in high-risk for psychosis populations,^45^ unmedicated first episode patients,^39,46,47^ and patients with chronic schizophrenia who are taking antipsychotic treatment,^48–51^ which suggests that resting state gamma abnormalities are present at illness onset^6,52,53^ and are not reversed by antipsychotic treatment. This is supported by other studies which have found no association between gamma power levels in schizophrenia and antipsychotic treatment^54,55^ or dose of treatment.^56^ Moreover, resting gamma power has been positively correlated with psychopathology^47,48,57^ and cognitive functioning in the disorder.^49,51,58^

Potassium Kv3.1 and 3.2 channels are highly expressed on parvalbumin positive interneurons,^59–61^ where they facilitate high-frequency action potential propagation.^62–65^ Modulation of Kv3.1 and Kv3.2 channels increases GABAergic interneuron firing frequency and improves gamma oscillation regularity.^66,67^ Potassium Kv3.1 and 3.2 channels have therefore been proposed as drug targets to restore parvalbumin interneuron function in schizophrenia.^68,69^ AUT00206 is a recently developed drug that modulates Kv3.1/3.2 channels.^70^ It has been shown to enhance whole-cell currents and the power of fast network oscillations,^71^ consistent with an action on parvalbumin positive interneurons. In addition, AUT00206 reversed the cognitive and social behavioural deficits induced by the NMDA-R antagonist phenyl cyclohexyl piperidine (PCP) in rodents, and enhanced gamma oscillations in human neocortical slices treated with PCP.^72^ However, AUT00206 has not been evaluated in schizophrenia to date. In view of the preclinical and clinical findings discussed above, we aimed to test two related hypotheses. The first was that resting gamma power would be directly related to psychotic symptom severity in patients early in the course of schizophrenia. The second was that AUT00206 would reduce frontal resting gamma power in patients with schizophrenia.

## Methods

We conducted this study using resting electroencephalography (EEG) to provide a test of mechanism and to address our hypothesis as part of a phase 1b study which had the main aim of evaluating the safety and tolerability of AUT00206 in schizophrenia. This paper reports on the resting EEG data only. The results of the safety and tolerability evaluations are reported elsewhere (ClinicalTrials.gov Identifier: NCT03164876). A placebo group was included for safety monitoring only and not for formal comparison with the AUT00206 group. However, we include the EEG results of the placebo group for qualitative comparison. The study protocol was approved by an NHS research ethics committee and appropriate authorities for all sites involved. The study was performed in accordance with the principles stated in the Declaration of Helsinki and Good Clinical Practice guidelines, as applicable at the time. All patients provided written informed consent before participation. The study was funded through the Innovate UK scheme and Autifony Ltd.

### Participants and Procedures

Patients were recruited from an NHS Trust in London between May 2017 and April 2019 to take part in the study. In total 24 patients with schizophrenia were randomised in a 2:1 ratio to receive repeated doses of either AUT00206 (16 subjects) or a placebo (PBO) (8 subjects). The participants were compensated for their time in the study.

Subjects randomised to active treatment received a loading dose of 2000 mg AUT00206 on Day 1, followed by repeated twice daily-oral doses of 800 mg AUT00206 on days 2–28 and a single oral dose of 800 mg AUT00206 on Day 28. Subjects randomised to placebo received placebo capsules that matched those of AUT00206 active capsules except minus the active, AUT00206. The initial loading dose was chosen to ensure blood levels of AUT00206 were within a target therapeutic range within the first 24 h, based on preclinical data (data on file, Autifony Therapeutics Ltd, Stevenage, UK). AUT00206 and antipsychotic levels were conducted throughout the study to monitor concordance.

Subjects underwent resting state ﻿electroencephalography (EEG) and positive and negative syndrome scale assessment (PANSS),^73^ at baseline, visits 1 and 2. Visits 1 and 2 were scheduled for days 6 and 28 after first dose, respectively, but could be brought forward in the event the subject was withdrawing early. The inclusion criteria were: male (due to a lack of safety data in females), 18–50 years of age, meeting criteria for schizophrenia (confirmed using the Structured Clinical Interview for DSM-5 Disorders, Clinician Version (SCID-5-CV)^74^), no more than 5 years to have passed since first diagnosis in order to reduce the confounding effect of time and long-term antipsychotic treatment on disease state; at least one positive symptom rating >3 or 2 or more positive symptoms rated = 3, and at least one negative symptom rating >3 or 2 or more negative symptoms rated = 3 on the PANSS; on a stable dose of 1 or 2 antipsychotic drugs (excluding clozapine) for at least 1 month before screening, and able to give fully informed written consent. No clinically relevant abnormalities on clinical examination or electrocardiography (ECG) or structural MRI findings were allowed.

Exclusion criteria were severely underweight or morbidly obese people, presence of an acute or chronic illness other than mild, well-controlled illnesses, homicidal ideation or intent, suicidal ideation with some intent to act in the last 6 months based on the Columbia-Suicide Severity Rating Scale^75^ (C-SSRS), moderate or severe depressive or anxiety symptoms as indicated by a score of ≥11 on the hospital anxiety and depression scale^76^ (HADS), presence or history of severe drug reaction, alcohol or drug dependence in the last 12 months before admission, presence of a contradiction to an MRI scan. Concomitant psychotropic medications were permitted, unless contraindicated due to their action on the cytochrome p450 (CYP) system.

### EEG Recording

EEG data were recorded on the Grass Comet System with a sampling frequency of 400 Hz and a built-in filter ([0.1, 100] Hz). Each lead was referenced to the Oz electrode. The maximum impedance was 5000 ohms. To reduce electromagnetic powerline noise, the system applies an in-built narrow 50 Hz (range 48–51 Hz) centred notch filter. EEG electrode location followed the 10–20 standard montage (as described in Ref. 77). To improve artefact identification, two electrooculographic (EOG) channels were recorded using silver cup electrodes.^78^ Resting-state EEG was recorded for at least 5 min, while participants were sitting in a chair with arm supports and their eyes closed in a suitably shielded room. Impedances were measured during all recordings and mean impedance was <2.5 kOhm in both groups (AUT00206 and PBO) at baseline and on treatment, with no significant differences found between groups at either time point (*P* > .05) using two-sample T-tests.

### EEG Analysis

The initial pre-processing stages were carried out in EEGLAB (version 2020)^79^ running on MATLAB.^80^ EEG data were cleaned using the EEGLab plugin *clean_rawdata* (version 2.2) without the artifact subspace reconstruction (ASR) algorithm,^81,82^ using the *clean_window* function only with default settings. In the eyes closed EEG signal, the major signal contaminant in the 35–45 Hz band is electromyographic activity (EMG).^83,84^ Large amplitude muscle activity has a characteristic readily identifiable high-frequency signal in the EEG.^85^ However, since EMG activity consists of many muscle fibre sources, a component-based correction algorithm, such as the Independent Component Analysis algorithm in EEGLAB, is not suitable for data with 20 EEG channels, as is the case here.^86^ Instead, data were inspected manually, blind to condition, and sections with high amplitude EMG activity which was spreading into the central midline locations, were excluded from analysis, using the EEGLab data scroll function.^87^ Gamma power, the primary frequency of interest, was extracted and quantified as the mean power (10 × log_10_ (*μ*V^2^/Hz)) in the 35–45 Hz band for each lead. Average gamma power (10 × log_10_ (*μ*V^2^/Hz)) was calculated for each subject at baseline, days 6 and 28.

### Electrode Derivations

**Figure 1.**
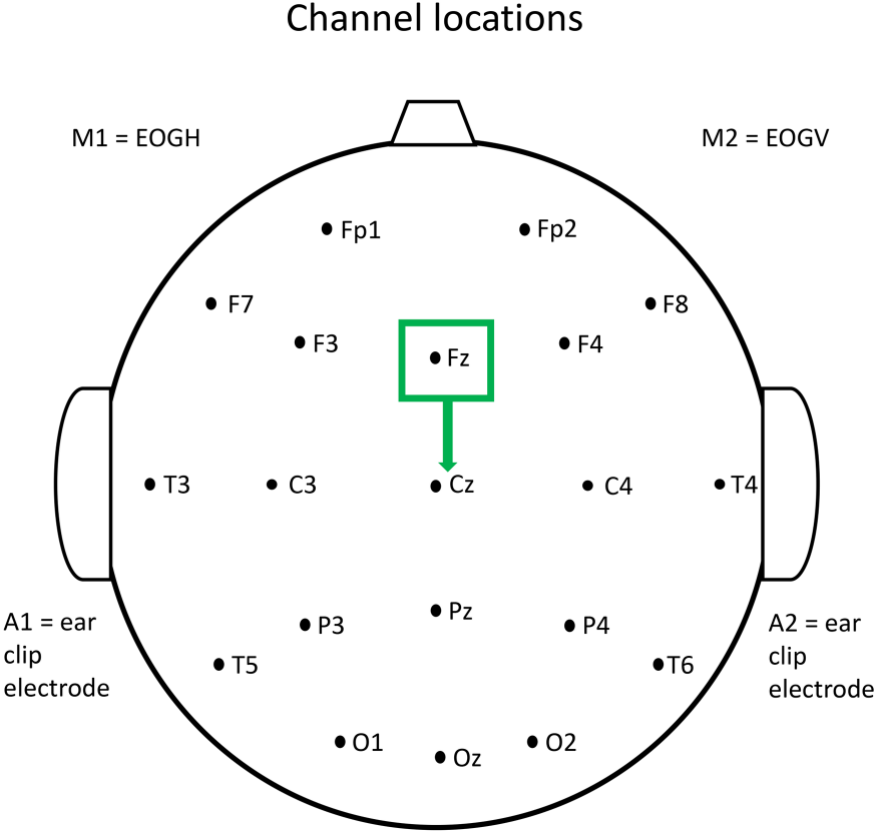
EEG scalp electrode location.

The signal from central midline electrodes, such as FZ and CZ, has been shown to have a low risk of electromyogenic (EMG) contamination,^88^ and only strong muscle contraction gives rise to contamination at these locations. FZ and CZ were therefore used for our analysis, with strong muscle contraction having been excluded. The FZ lead was re-referenced to CZ to give a measure of frontal activity as shown in Figure 1.

### Statistical Analysis

The frontal region (FZ) was our primary region of interest based on extensive data indicating this is a key brain region implicated in the pathophysiology of schizophrenia.^89^ Statistical analysis was carried out using SPSS statistical analysis software.^90^ Frontal resting state gamma amplitude (*μ*v/Hz) was not normally distributed (Kolmogorov–Smirnov and Shapiro–Wilk tests of normality <0.05), so the EEGLab output power data (10 × log_10_ (*μ*V^2^/Hz)), were used in the primary analyses, which passed tests of normalcy. To test our first hypothesis that there is a direct relationship between frontal resting state gamma power and symptom severity, we correlated resting state gamma power (10 × log_10_ (*μ*V^2^/Hz)) in the whole sample with PANSS positive symptom scores at baseline using Pearson's product moment correlation. To test our hypothesis that treatment with AUT00206 would reduce frontal resting gamma power, we used a Students Paired t-test to determine if there was a significant change in mean on-treatment (days 6 and 28) frontal gamma power (10 × log_10_ (*μ*V^2^/Hz)) versus baseline gamma power in the AUT00206 group. We include the results of the placebo group for reference only, as the study was not powered to find a difference between groups. However, we also conducted an exploratory analysis to determine if there was an indication of a group difference in gamma-band power (10 × log_10_ (*μ*V^2^/Hz)) change following treatment. We calculated two ‘gamma power (10 × log_10_ (*μ*V^2^/Hz)) change’ indices:

(1) Δgammapower(d6)=gammapower(d6)−gammapower(baseline)

(2) Δgammapower(d28)=gammapower(d28)−gammapower(baseline)

To test group difference and the effects of co-variants, an analysis of covariance (ANCOVA) was conducted using Δgammapower(d6)andΔgammapower(d28) for each participant as dependent variables. Treatment group (AUT00206 or PBO) was included as a fixed factor, and age (years), daily equivalent chlorpromazine dose (mg/day) and baseline gamma power (10 × log_10_ (*μ*V^2^/Hz)), as co-variates, due to evidence that oscillatory activity changes with age,^91,92^ that there may be an effect of antipsychotic medication on electrophysiological activity^93^ including the gamma-band^94^ and to account for regression to the mean. This was corrected for multiple comparisons using the Bonferroni correction. We additionally conducted a spectral analysis in EEGLab using a repeated measure analysis of variance (ANOVA) in the AUT00206 and PBO groups with time (baseline, days 6 and 28) as a fixed factor and Bonferroni correction to determine if there were significant changes in resting power over time in either group. Baseline clinical and demographic comparisons between groups were calculated using either Chi square (categorical data) or Students t-test (continuous data).

## Results

### Demographics and Clinical Data

Table 1 shows the demographic and clinical characteristics of the final EEG study population at baseline (*n* = 22, AUT00206 *n* = 14, PBO *n* = 8) following the exclusion of two patients with unusable EEG data due to excessive movement artefacts. There were no significant differences between the treatment and placebo groups in terms of age, ethnicity, antipsychotic dose, or baseline symptoms rating or illness severity. All patients received their assigned treatment from days 1 to 28, except one patient in the AUT00206 group, who received his treatment from days 1 to 21 only as he dropped out of treatment after this. His final EEG was taken on day 21. On PK sampling the median Cmax for AUT00206 was 3745 ng/mL and the mean Ctrough (Cpre-dose) values were >2300 ng/mL on days 4 to 6, ∼1800 ng/mL on day 14 and ∼2200 ng/mL on days 21 and 28. Target concentrations, based on preclinical models and the ketamine-challenge study in HVs, were between 1500 and 4000 ng/mL.

**Table 1.** Baseline Demographic and Clinical Characteristics of the EEG Population. CPZ = Chlorpromazine Equivalent Daily Dose, PANSS = Positive and Negative Symptom Scale, CGI = Clinical Global Impression Scale.

Demographic and clinical characteristics of EEG population at baseline			
	AUT00206	PBO	
	*n* = 14	*n* = 8	
Male *n*(%)	14 (100)	8 (100)	
Ethnicity: black *n*(%)	12 (86)	5 (63)	
Ethnicity: white *n*(%)	2 (14)	2 (25)	
Ethnicity: other *n*(%)	0 (0)	1 (13)	*P* = .3
Age years mean(sd)	27.6 (6.3)	28.7 (5.2)	*P* = .28
CPZ equi/day mean(sd)	238.4 (143.6)	180.8 (37.1)	*P* = .18
Baseline PANSS total mean(sd)	77.1 (10.1)	75.6 (7.8)	*P* = .72
Baseline PANSS positive mean(sd)	19.1 (4.2)	18.7 (1.8)	*P* = .84
Baseline PANSS negative mean(sd)	20.2 (3.5)	19.6 (3.9)	*P* = .77
CGI mean(sd)	3.6 (0.6)	3.8 (0.5)	*P* = .81

### Correlation between Baseline Frontal Gamma Power and Symptoms

**Figure 2.**
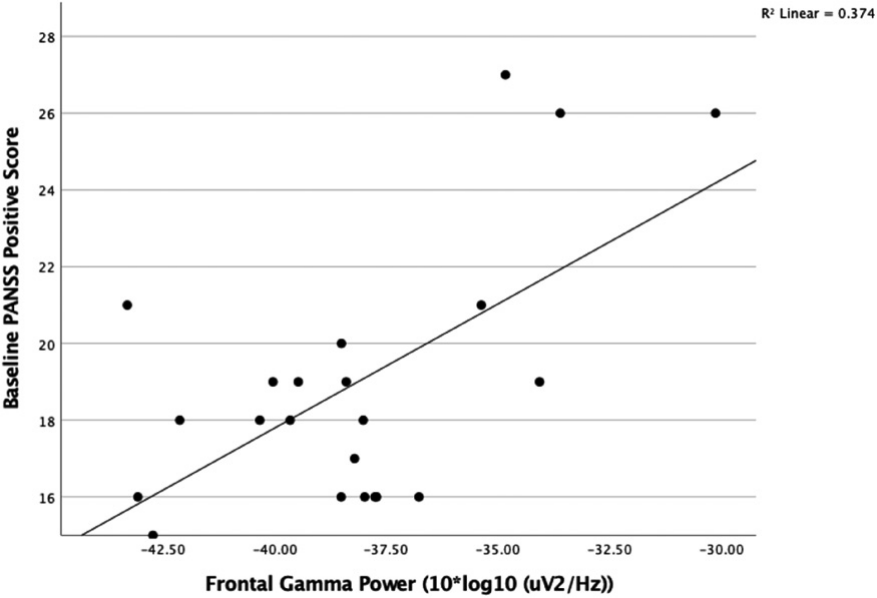
There was a positive correlation between frontal resting gamma power (10 × log_10_ (*μ*V^2^/Hz)) with baseline PANSS positive symptom severity score (*n* = 22, *r* = 0.613 *P* < .002).

At baseline, we found a significant positive correlation between resting gamma power (35–45 Hz band) in the frontal region (*n* = 22, *r* = 0.675, *P* < .001) and PANSS positive severity score (Figure 2). There was no correlation between CPZ dose or age and baseline gamma power (*P* > .1) There was no correlation with total or negative symptom severity scores.

### Change in Frontal Gamma Power

**Figure 3.**
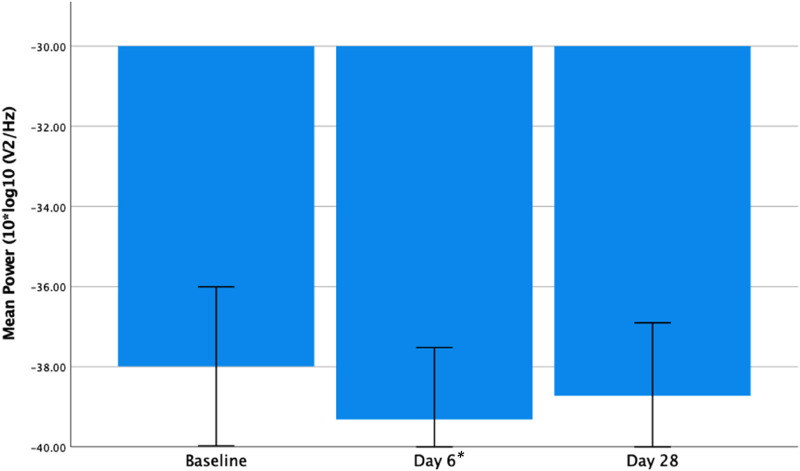
Frontal resting gamma power (10 × log_10_ (*μ*V^2^/Hz)) at baseline, days 6 and 28 in the AUT00206 group. Showing a significant reduction from baseline to day 6 (*t*_13_ = 3.635, *P* = .003) and a trend significant reduction from baseline to day 28 (*t*_13_ = 1.961, *P* = .072). Error bars = 95% confidence intervals. *P < .05.

**Figure 4.**
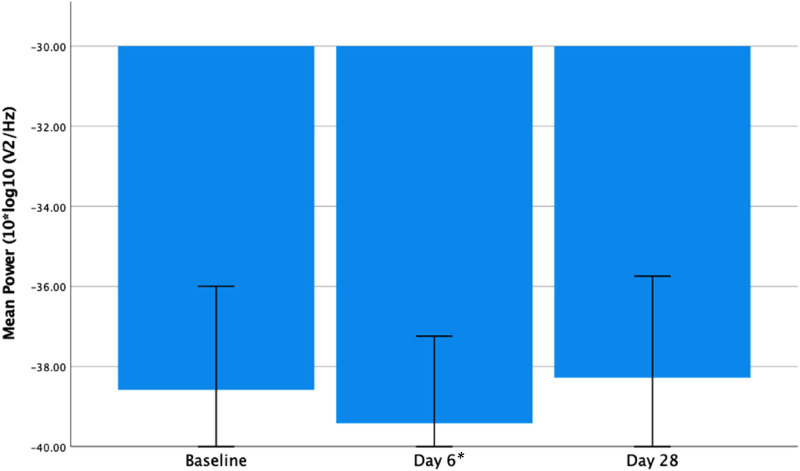
Frontal resting gamma power (10 × log_10_ (*μ*V^2^/Hz)) at baseline, days 6 and 28 in the PBO group. Showing a significant reduction from baseline to day 6 (*t*_7_ = 2.626, *P* = .035) and non-significant increase from baseline to day 28 (*t*_7_ = −0.564, *P* = .591). Error bars = 95% confidence intervals. *P < .05.

There was a significant reduction in frontal gamma power from baseline to day 6 (*t*_13_ = 3.635, *P* = .003) and a trend for a reduction at day 28 relative to baseline (*t*_13_ = 1.961, *P* = .072) (see Figure 3 below). A within group analysis of PBO subjects also found a significant reduction in frontal gamma power from baseline to day 6 (*t*_7_ = 2.626, *P* = .034) on paired t-test. This had reversed at day 28, showing an increase, but this was not significantly different from baseline (*t*_7_ = −0.564, *P* = .591) (see Figure 4 below).

**Figure 5.**
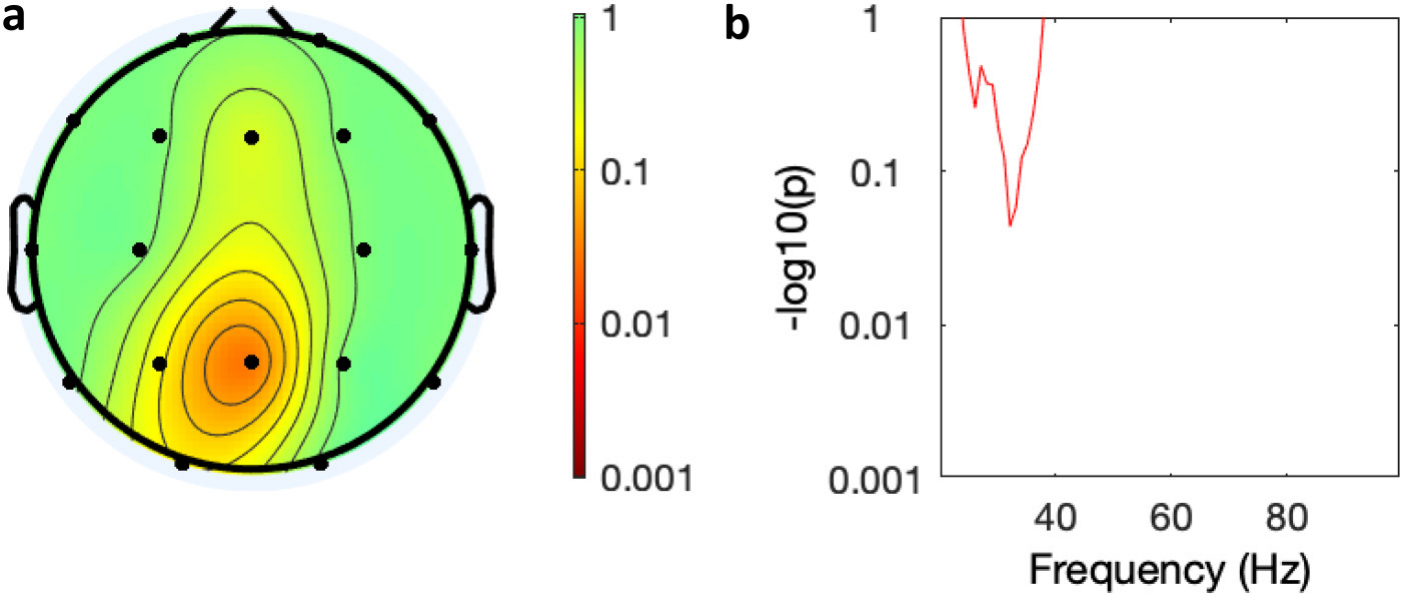
Spectral analysis of the effect of time on power in the AUT00206 group. (a) Topology of average power change across all leads over time (baseline, days 6 and 28), (colour bar = *P*-value Bonferroni corrected); (b) graphical display of effect of time on power over frequency in the Fz lead (*y* axis = *P*-value Bonferroni corrected).

**Figure 6.**
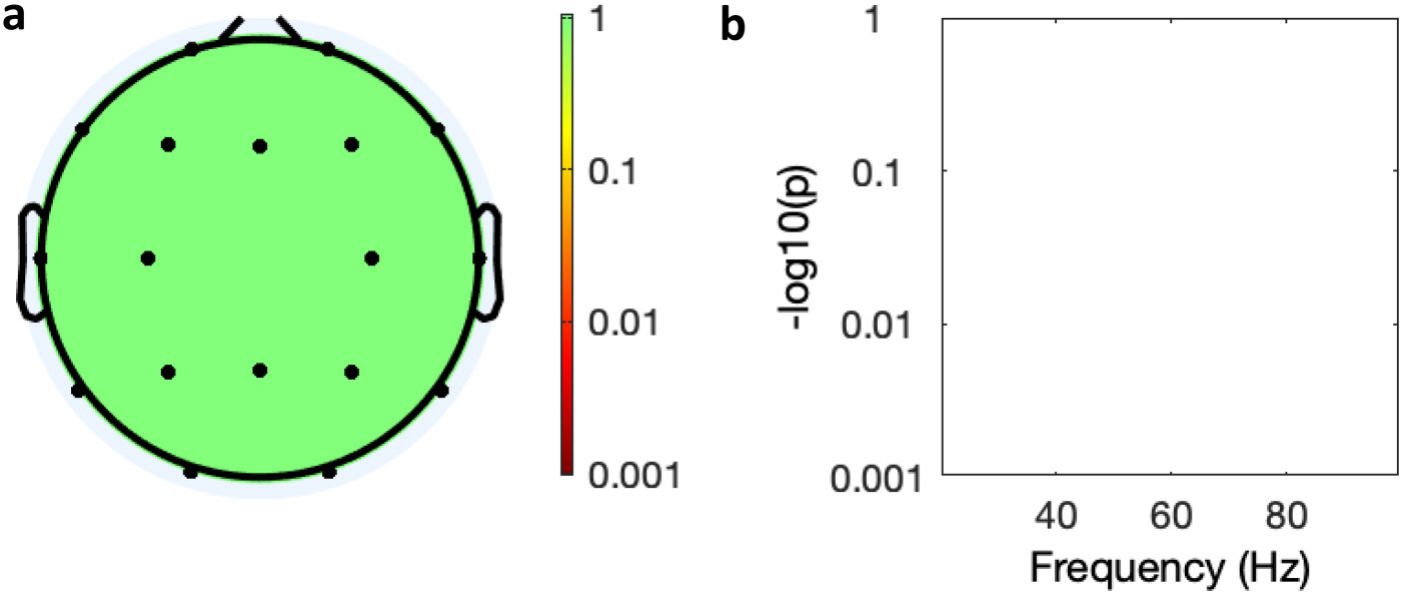
Spectral analysis of the effect of time on power in the PBO group. (a) Topology of average power change across all leads over time (baseline, days 6 and 28), (colour bar = *P*-value Bonferroni corrected); (b) graphical display of effect of time power over frequency in the Fz lead (*y* axis = *P*-value Bonferroni corrected).

In the exploratory analysis of change in gamma power between treatment groups (AUT00206 and PBO), there was no significant effect of age, chlorpromazine equivalent dose, baseline gamma power, or treatment group on Δgammapower(d6) . However, there was a significant effect of age (*F* = 6.161, *P* = .024, chlorpromazine dose (*F* = 5.086, *P* = .038), baseline gamma power (*F* = 9.380, *P* = .007), and trend effect of treatment (*F* = 3.745, *P* = .07) on Δgammapower(d28) . In the ANOVA of change in spectral power over time (baseline, days 6 and 28) there was a trend reduction in the AUT00206 group in frontal, central and posterior regions (Figure 5) that was not present in the PBO group (Figure 6).

## Discussion

This study found that higher resting state gamma power in frontal regions correlates with more severe positive symptoms of schizophrenia and that treatment with AUT00206 over 6 days was associated with a significant reduction in resting frontal gamma power in patients. Similar findings were seen in the frontal region in the PBO group at day 6, however, there was a trend increase by day 28 in the PBO group that was not seen in the AUT00206 group. The positive correlation between frontal resting gamma power and the positive symptoms of psychosis extends previous work showing positive correlations between symptom severity and gamma band power in chronic schizophrenia^48^ to show this in patients within 5 years of illness onset. AUT00206 has been shown to reduce BOLD signal changes in cortical regions in healthy volunteers given ketamine as a model of the excitation-inhibition balance seen in schizophrenia.^95^ Our findings extend this, by providing evidence that it reduces resting gamma power, thought to reflect excitation-inhibition imbalance, in schizophrenia for the first time.

### Strengths and Limitations

Strengths include this being the first study of AUT00206 in people with schizophrenia and the selection of a patient cohort early in the course of illness. The main limitations are the modest sample size and lack of a healthy control group to demonstrate whether gamma power was higher in the patients. The sample size was limited due to safety and logistic considerations because it was the first study in the disorder. The lack of a significant reduction in frontal power at day 28 may be due to the study being underpowered to detect more subtle, sustained change given that the effect showed a trend towards significance. A change in the placebo group was not expected though this could represent a regression to the mean or a chance finding. However, regression to the mean was accounted for in our ANCOVA analysis which used baseline values as a co-variant and found a trend (*P* = .07) difference in gamma power change at day 28 between treatment groups. Furthermore, the spectral analysis ANOVA findings indicate a change in power over treatment time in the AUT00206 group that is not seen in the PBO. In addition, as gamma activity is related to brain activation it can vary with the level of arousal or relaxation, and so a larger placebo group would have enabled us to control for any systematic day related changes in the participant's psychological state. Thus, the findings warrant further evaluation in a larger sample, including a greater number treated with placebo to increase the power for comparisons with placebo. Our sample also consisted entirely of men due to the safety requirements of the main study. This could limit the generalisability of our findings and highlights the need for a further study including females. The notch filter precluded an analysis of the gamma range between 48–51 Hz. However, our analysis includes typical gamma frequency bands consistent with prior studies in schizophrenia.^34^ All patients in this study were treated with concomitant antipsychotic drugs. However, previous studies have not linked resting state gamma with antipsychotic dosage^56^ and antipsychotic treatment was stable prior to enrolment and between resting state EEG measurements, suggesting this is unlikely to be a major confound.

### Interpretation and Implications

Our findings advance previous evidence that excitatory-inhibitory imbalance is implicated in the pathophysiology of schizophrenia^96,97^ by showing frontal gamma oscillation power correlates with positive (psychotic) symptoms and that AUT00206 may target this mechanism, unlike current antipsychotics.^2^ AUT00206 is a Kv3.1/3.2 channel modulator^98^ that enhances fast network oscillations (Large et al., 2016). Kv3.1/3.2 channels are highly expressed on parvalbumin interneurons,^60,99,100^ where they are responsible for allowing such neurons to propagate action potentials at the high rates seen during gamma frequency activity.^62–65^ Furthermore a Kv3.1 knockout model showed increased gamma power^101^ and unmedicated patients with schizophrenia are known to have reduced Kv3.1 expression,^102^ and increased resting gamma power.^49^ Thus, the action of AUT00206 on Kv3.1/3.2 channels could explain our finding that AUT00206 reduces frontal power in the disorder. Finally, in rodents, AUT00206 reversed the cognitive and behavioural effects of phencyclidine (PCP),^103^ suggesting that it could target cognitive and other symptoms of the disorder. Our findings extend the preclinical evidence by showing a relationship between frontal gamma power change and positive symptom change. These findings thus provide initial evidence of the drug acting on a mechanism relevant to a key component of the pathophysiology of schizophrenia and support the evaluation of AUT00206 in larger clinical studies to determine its potential to ameliorate symptoms.

## Conclusions

We found that resting gamma power correlated with PANSS positive score at baseline in patients early in the course of schizophrenia, with higher resting power associated with more severe positive symptoms. In the group treated with AUT00206 there was a reduction in frontal gamma power following treatment. These findings identify an association between gamma oscillations and positive symptoms consistent with impaired GABAergic signalling in schizophrenia and suggest that the potassium channel modulator AUT00206 could address a key aspect of the pathophysiology of schizophrenia. Further studies should investigate the potential of Kv3.1/3.2 modulation as a novel treatment target in the disorder.
